# Instability motion characteristics of overburden rock and the distribution pattern of fissures in shallow thick seam mining

**DOI:** 10.1038/s41598-022-10205-z

**Published:** 2022-04-13

**Authors:** Jianwei Li, Changyou Liu, Xinwei Guo, Xiangye Wu

**Affiliations:** 1 Institute of Mining Engineering, Inner Mongolia University of Science and Technology, Baotou, 014010 Inner Mongolia China; 2 School of Mines, China University of Mining and Technology, Xuzhou, 221116 Jiangsu China

**Keywords:** Civil engineering, Geodynamics

## Abstract

This paper analyzes the instability movement characteristics of overburden in shallow thick coal seam mining and its influence on the development and distribution of fault fractures. The similarity simulation experiment and theoretical analysis were combined based on the classification of the occurrence characteristics of the key bearing layer in the overburden rock of shallow thick seam mining. This study investigated the fracture characteristics and the instability motion mode of the key bearing layer in shallow thick seam mining and their effects on the distribution of fissures in the overburden rock. The results indicated that according to the horizon of the key bearing layer, the occurrence of overburden rock could be classified into 2 categories, i.e., the horizon of the key bearing layer within the caving zone and within the fissure zone. The horizon of the key bearing layer has a significant effect on the fracture characteristics and the instability motion mode of the key bearing layer. When the horizon of the key bearing layer is in the overburden caving zone, a "step rock beam" develops after fracture, and the instability motion mode is sliding instability. When the horizon of the key bearing layer is in the overburden fissure zone, a "masonry-like beam" develops after fracture, and the instability motion mode is rotary instability. The fracture instability of the key bearing layer could control the development and distribution of fissures in the overburden rock, and the whole favorable zone for the development of fissures extends along the advancing direction of the working face in a form of "diagonal stripes" with the instability motion of the key bearing layer.

## Introduction

As the focus of coal resource development in China shifts further to the west, especially due to the continuous escalation of the shallow coal seam mining scale represented by the Shendong Mining Area, the western region has become the principal source of coal in China^1–3^. Using Shendong Mining Area as an example, the mined seams are principally concentrated in the Yanan Formation of the Jurassic System. The buried depth of the coal seam is ≤ 150 m, which belongs to the shallow buried coal seam, and the overlying rock is relatively thin. The surfaces covered with loess or aeolian sand, and fragile ecological surface environments^4–7^.

Shallow coal seams can cause serious surface damage, mining subsidence, and fissures during mining^8,9^. The surface collapses and fissures connected to the underground goaf area may frequently cause safety hazards, such as air leaks and water bursting-induced sand collapses on the working face, as well as the loss of groundwater resources and destruction of the surface ecology, which can affect safe and ecologically friendly mining in mines^10–12^. The typical mining strata pressure behaviors of shallow coal seams are as follows. The fracture and instability motion of the key bearing layer in the bedrock layer directly result in obviously higher resistance at stope supports, support damage, rib spalling, and sidestepped subsidence and may cause overall movement of the surface overburden, possibly causing mining fissures directly connected to the surface^13–16^. Therefore, the fracture instability of the key bearing layer has a controlling effect on the intensity of mine strata pressure behaviors on the working face and the development and distribution of fissures^17,18^.

Based on classification of the occurrence characteristics of the key bearing layer in the overburden rock of shallow thick seam mining, this paper combined similarity simulation experiment and theoretical analysis to study the fracture characteristics and instability motion mode of the key bearing layer for shallow thick seam mining and their effects on the distribution of fissures in overburden rock. The study findings are of certain guidance and reference significance to the surface subsidence of shallow thick seams, the coordinated control of the mine strata pressure on the working face, the water bursting-induced sand collapse in the goaf area, and the spontaneous combustion of leftover coal.

## Classification of the occurrence characteristics of the overburden rock of shallow thick seam mining

According to previous research results^19,20^, there is a key bearing rock layer in the overlying rock layer of the coal seam. The periodic fracture of this rock stratum causes movement of its overlying strata and the loose layer and leads to ground ground surface fissures. This stratum is called key bearing layer. The key bearing layer is a hard and thick stratum, which supports the upper strata and the loose covering layer in some form of mechanical structure. Their periodic fracture directly influences roof pressure, strata movement, and mining subsidence. Therefore, studying the movements of the key bearing layer is of great significance for instability motion characteristics of the overburden rock and their effects on the development and distribution of fissures.

**Figure 1:**
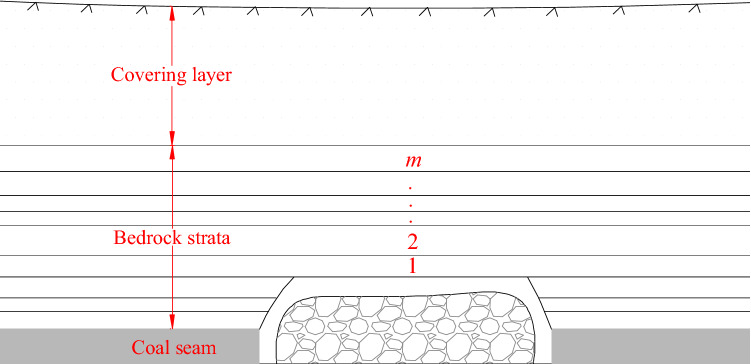
Distribution of overlying strata in shallow thick coal seam.

The distribution of the overlying strata in the shallow coal seam is shown in Fig. 1. The coal seam is covered with strata of 1 to *m*. The upper layer is the loose covering layer which is composed of soil or weak rock^21–23^.

The thickness of each strata is set to be *h*_*i*_, bulk density is *γ*_*i*_, elastic modulus is *E*_*i*_, and *i* = l, 2, 3, …, *m*. According to the theory of key strata, the loading key strata of overlying strata in shallow coal seam must meet the following three conditions^24^:

1 $$\left\{ \begin{aligned} & q_{n} > q_{n - 1} > \cdots > q_{1} \hfill \\ & q_{n} = (q_{n} )_{overburden} , {\text{and}} (q_{n} )_{overburden} > (q_{n} )_{n + 1} > \cdots > (q_{n} )_{m} \hfill \\ & L_{n} > L_{n + 1} > \cdots > L_{m} . \hfill \\ \end{aligned} \right.$$

Among them:

$$\left\{ \begin{aligned} & (q_{n} )_{overburden} = {{E_{{\text{n}}} h_{n}^{3} \cdot \left( {\sum\limits_{i = n}^{i = m} {\gamma_{i} h_{i} } + \gamma_{overburden} h_{overburden} } \right)} \mathord{\left/ {\vphantom {{E_{{\text{n}}} h_{n}^{3} \cdot \left( {\sum\limits_{i = n}^{i = m} {\gamma_{i} h_{i} } + \gamma_{overburden} h_{overburden} } \right)} {\sum\limits_{i = n}^{i = m} {E_{i} h_{i}^{3} } }}} \right. \kern-\nulldelimiterspace} {\sum\limits_{i = n}^{i = m} {E_{i} h_{i}^{3} } }} \hfill \\ & (q_{n} )_{m} { = }{{E_{n} h_{n}^{3} \cdot \sum\limits_{i = n}^{i = m} {\gamma_{i} h_{i} } } \mathord{\left/ {\vphantom {{E_{n} h_{n}^{3} \cdot \sum\limits_{i = n}^{i = m} {\gamma_{i} h_{i} } } {\sum\limits_{i = n}^{i = m} {E_{i} h_{i}^{3} } .}}} \right. \kern-\nulldelimiterspace} {\sum\limits_{i = n}^{i = m} {E_{i} h_{i}^{3} } .}} \hfill \\ \end{aligned} \right.$$

In the formula:

- (*q*_*n*_)_*overburden*_—the load of bedrock strata and covering layer upon strata *n*, kN/m^2^;
- (*q*_*n*_)_*m*_—the load of strata *m* upon strata *n*, kN/m^2^;
- *q*_*i*_ (*i* = 1, 2, 3, …, *n*)—the load of the stratum *i*, kN/m^2^;
- *L*_*i*_ (*i* = *n*, *n* + 1, …, *m*)—the caving step of the stratum *i*, m;
- *γ*_*i*_, *E*_*i*_, *h*_*i*_—the average bulk density, elastic modulus and thickness of stratum *i*, kN/m^3^, MPa, m;
- *γ*_*overburden*_, *h*_*overburden*_—the average bulk density and thickness of bedrocks and overburden, kN/m^3^, m.

The position and thickness of the key bearing layer in the overburden rock of a mining area not only affect the fragment dimensions and instability motion mode but also can significantly affect the instability motion and the distribution of mining-induced fissures in the overburden rock of shallow thick seam mining. Therefore, the occurrence conditions of the key bearing layer in stope overburden rock were classified based on the typical occurrence conditions in overburden rock of shallow thick seam working faces in Western China, setting a foundation for further analysis of the fracture instability of overburden rock in shallow thick seam mining and the distribution patterns of fissures.

Table 1 shows the statistical analysis of the information about working face seam thickness and the horizon and thickness of the key bearing layer in shallow thick seam mining in Western China.

**Table 1:** Statistics of the overburden rock occurrence characteristics of shallow thick seam mining in Western China (the rock hulking coefficient in the caving process is 1.25).

Coal Mine	Key bearing layer lithology	Coal seam occurrence condition		Horizon and thickness of key bearing layer		Height/m	Position of key bearing layer
		Seam thickness/m	Seam depth/m	Distance from coal seam/m	Thickness/m		
Da Liuta coal mine	Fine sandstone	14.6	102.3	38.3	10.3	58.2	Caving zone
Bu Ertai coal mine	Sandy mudstone	17.7	145.8	75.1	16.3	70.8	Fissure zone
Chang Hangou coal mine	Medium sandstone	17.9	110.2	76.3	32.2	71.6	Fissure zone
Liu Ta coal mine	Fine sandstone	9.2	90.9	30.5	15.8	36.9	Caving zone
Cun Caota coal mine	Sandy mudstone	7.8	146.2	45.1	26.3	31.3	Fissure zone
Shang Wan coal mine	Sandy mudstone	7.2	64.4	26.6	14.4	28.9	Caving zone
Ha Lagou coal mine	Fine sandstone	5.5	94.4	49.3	18.5	22.0	Fissure zone
Yu Jialiang coal mine	Fine sandstone	14.9	140.3	81.6	31.9	59.5	Fissure zone
Chuancao Gedan coal mine	Siltstone	12.8	116.6	63.4	9.2	51.2	Fissure zone

**Figure 2:**
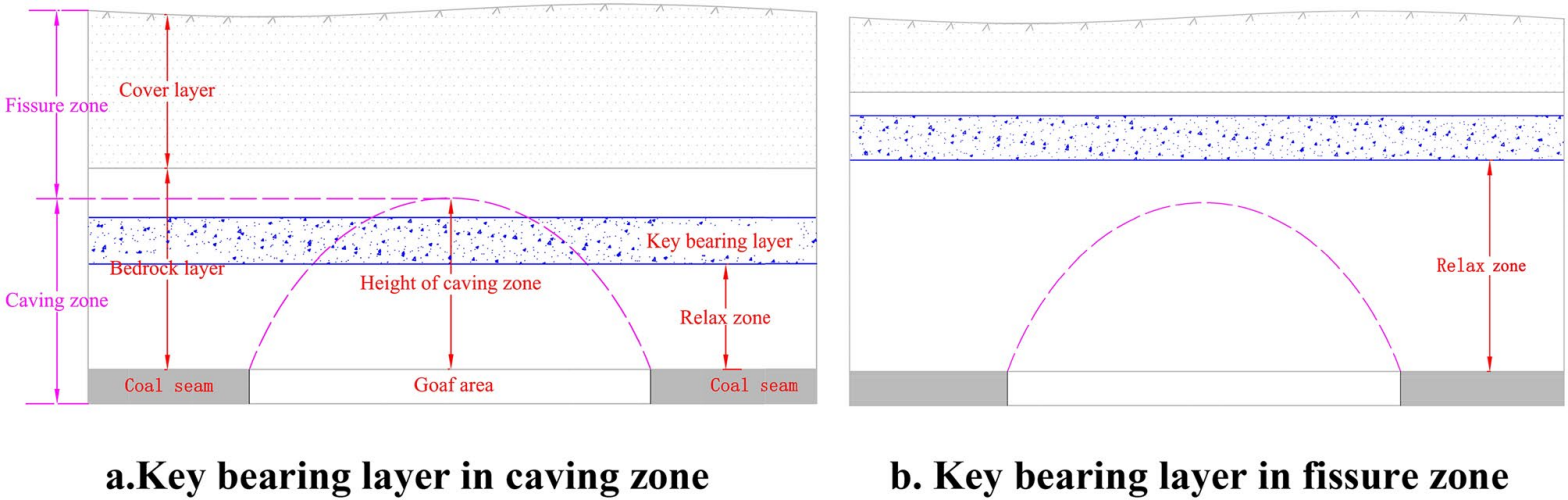
Classification of the occurrence conditions in the overburden rock for shallow thick seam mining.

The occurrence conditions of the key bearing layer in the overburden rock in shallow thick seam mining were classified into two categories by the horizon of the key bearing layer in the overburden rock with reference to the typical occurrence conditions of the shallow thick seam working face in China, i.e., the key bearing layer in the overburden caving zone (Fig. 2a) and the key bearing layer in the overburden fissure zone (Fig. 2b). The rock stratum under the key bearing layer above the roof of the excavation is defined as a relaxed zone^25^, as shown in Fig. 2.

## Building of a physical similarity simulation model and experimental schemes

Based on the occurrence conditions of coal and rock layers of shallow thick coal seam mining in a coal mine in western China, physical similarity simulation testing on the plane stress was performed to analyze the fracture instability characteristics of overburden rock with an advancing working face when the key bearing layer of shallow thick seam mining is in the overburden caving zone and fissure zone.

**Figure 3:**
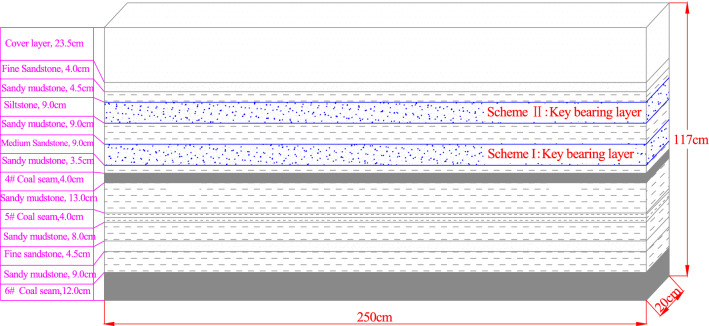
Similarity simulation model.

The size of the physical similarity simulation test stand was L × W × H = 2500 mm × 200 mm × 2000 mm. According to the theory of similarity, the geometric similarity ratio was 1:100, the bulk density similarity constant was 1.56, the stress similarity constant was 156 and the time similarity constant was 10^26^. The similarity simulation models built for the key bearing layer located in the overburden caving zone and the fissure zone when the 4# seam was mined are shown in Fig. 3. According to scheme I in the figure, the key bearing layer of the overburden rock was 9.0 cm-thick medium-grained sandstone when the 4# seam was mined, and the height of the relax zone is 3.5 cm. According to scheme II, the key bearing layer was 9.0 cm-thick siltstone when the 4# seam was mined, and the height of the relax zone is 21.5 cm. In the similarity simulation test, the simulation materials for each rock formation were made from sand, calcium carbonate, lime, gypsum, and water mixed in certain proportions. The materials were horizontally arranged in layers, and the mica powder was spread between the layers to simulate the weak planes of the rock. For rock strata greater than 5.0 cm thick (except for the key bearing layer), a layered arrangement was performed according to the actual distribution of the weak planes. The compressive strength was taken as the principal similarity condition, and the similarity criteria were met during the modeling. The model does not require additional loads. Table 2 shows the physical and mechanical parameters of each layer.

**Table 2:** Physical and mechanical parameters of each layer.

Serial number	Lithology	Thickness/m	Burial depth/m	Density/kg m^−3^	Compressive strength/MPa	Tensile strength/MPa	Notes
1	Cover layer	23.5	23.5	2250	–	–	
2	Fine sandstone	4	27.5	2595	51.3	3.2	
3	Sandy mudstone	4.5	32	2550	33.5	1.3	
4	Siltstone	9	41	2650	54.9	3.0	Scheme II
5	Sandy mudstone	9	50	2550	34.9	1.1	
6	Medium sandstone	9	59	2500	45.2	2.3	Scheme I
7	Sandy mudstone	3.5	62.5	2550	32.5	1.5	
8	4# coal seam	4	66	1450	14.6	1.32	
9	Sandy mudstone	13	79	2550	28.9	1.3	
10	5# coal seam	4	83	1450	14.2	1.32	
11	Sandy mudstone	8	91	2550	32.5	1.5	
12	Fine sandstone	4.5	95.5	2595	58.4	3.0	
13	Sandy mudstone	9	104.5	2550	34.9	4.1	
14	6# coal seam	12	116.5	1450	16.1	1.3	

After the model is made, parallel and vertical survey lines are arranged in the overall model, the spacing of survey lines is 10 cm, and survey points are arranged at the intersection of survey lines. During the simulation of excavation in the 4# coal seam, the mining height was 4.0 cm, the excavation step was 5.0 cm, and the length of the working face was 190 cm. For each excavation, the three-dimensional photogrammetry system is used to record the number of meters excavated in the working face and the data on the change in displacement of the overburden.

## Fracture instability characteristics of overburden rock in shallow thick seam mining

### Key bearing layer within the overburden caving zone

**Figure 4:**
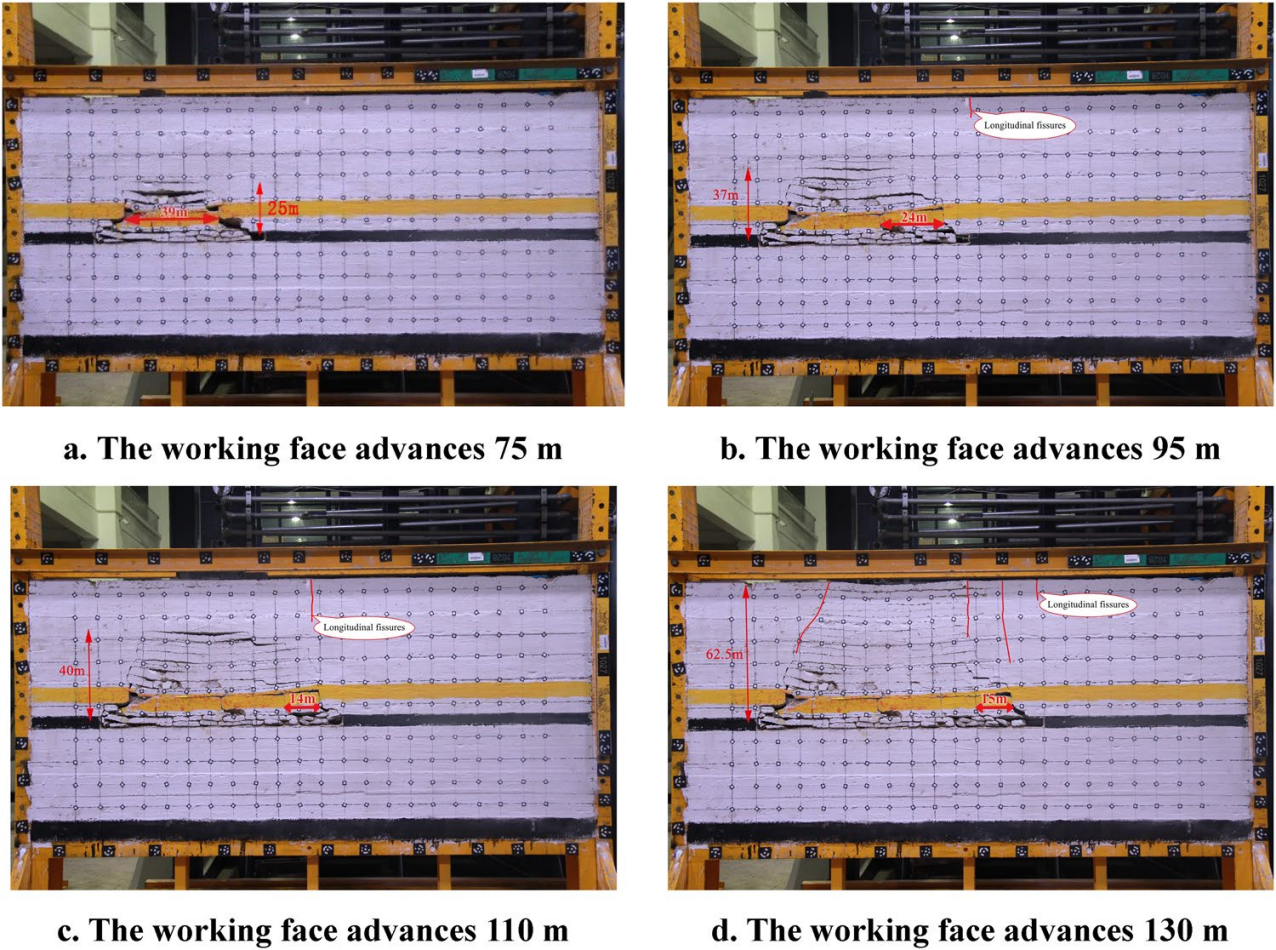
Overburden rock instability characteristics and fissures distribution characteristics of the key bearing layer located within the caving zone.

When the key bearing layer is within the overburden caving zone for 4# coal seam mining, the fracture instability characteristics of the overburden rock and the distribution characteristics of the fissures at different advancing distances are shown in Fig. 4.

As shown in Fig. 4, when the working face of the 4# coal seam advances approximately 75 m, the 9.0 m-thick medium-grained sandstone key bearing layer develops initial fracture weighting, and the length of fractured rock is 39 m. The fracture and instability motion of the key bearing layer cause the overburden rock to collapse and develop horizontal separation fissures. The vertical extent of the destruction zone is 25 m.

When the working face of the 4# coal seam advances approximately 95 m, the 9.0 m-thick medium-grained sandstone key bearing layer develops the first periodic fracture weighting, the fracture is located at the coal wall of the working face, and the length of the fractured rock is 24 m. The height of caving zone of the overburden rock increases, and the vertical extent of the destruction zone is 37 m. Longitudinal cracks appear on the ground surface.

When the working face of the 4# coal seam advances approximately 110 m. The vertical extent of the destruction zone is 40 m. The fracture depth from the ground surface downwards increases to approximately 17 m, but the ground surface fracture does not connect with the fissures in the overburden rock.

When the working face of the 4# coal seam advances approximately 130 m. The vertical extent of the destruction zone is 62.5 m. Multiple ground surface fractures and fissures in the overburden rock are connected. Significant subsidence on the ground.

### Key bearing layer within the overburden fissure zone

**Figure 5:**
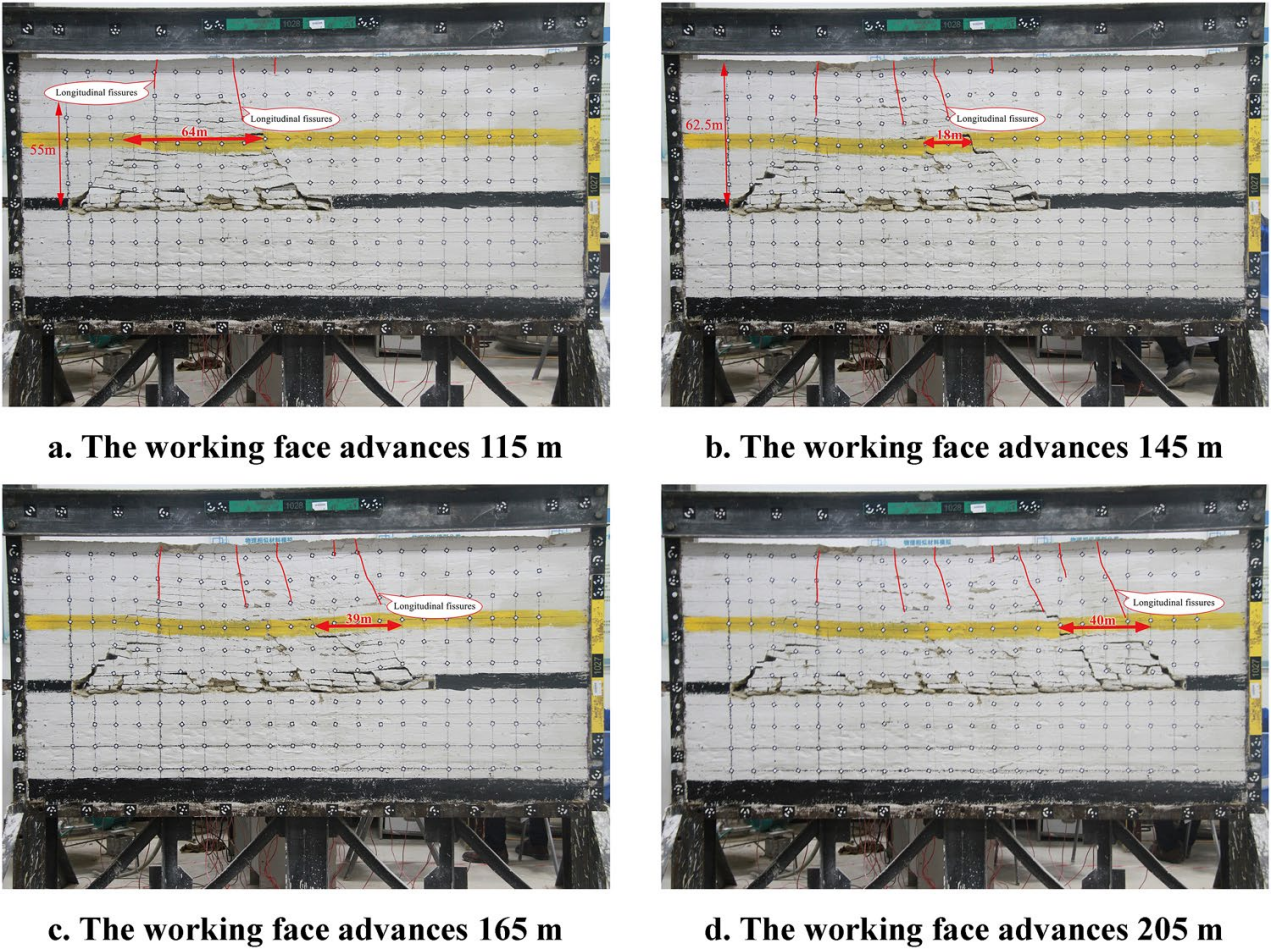
Overburden rock instability characteristics and fissures distribution characteristics of the key bearing layer located within the fissure zone.

The fracture instability characteristics of the overburden rock and distribution characteristics of the fissures at different advancing distances when the key bearing layer is within the overburden fissure zone for 4# coal seam mining are shown in Fig. 5.

As shown in Fig. 5, when the working face of the 4# coal seam advances approximately 115 m, the 9.0 m-thick siltstone key bearing layer develops initial fracture weighting, and the length of the fractured rock is 64 m. The fracture and instability motion of the key bearing layer cause the overburden rock to collapse and develop horizontal separation fissures and longitudinal fissures connected to the ground surface. The vertical extent of the destruction zone is 55 m.

When the working face of the 4# coal seam advances approximately 145 m, The vertical extent of the destruction zone is 62.5 m. Multiple ground surface fractures and fissures in the overburden rock are connected. Significant subsidence on the ground.

When the working face of the 4# coal seam advances approximately 165 m, the 9.0 m siltstone key bearing layer develops the second periodic fracture weighting, and the length of fractured rock is 39 m. The fracture movement causes longitudinal fissures directly connected to the ground surface to develop in the overburden rock and the cover layer, and the mean distance from other longitudinal fissures connected to the ground surface is approximately 30 m.

After, during the advancing process of the working face of the 4# coal seam, the number of ground surface fissures has increased. The ground surface subsides periodically.

### Fracture instability motion mode of the overburden rock

**Figure 6:**
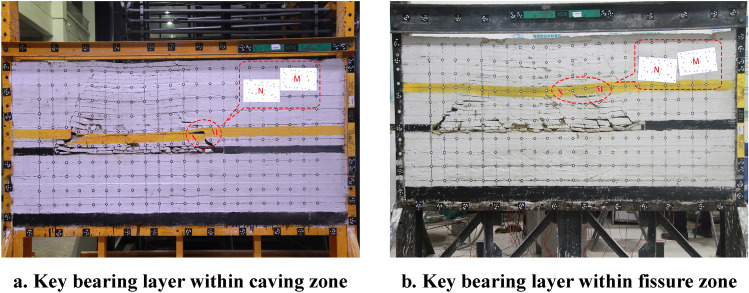
Comparison of the periodic fracture instability motion modes of the key bearing layer and movement characteristics of overburden rock.

According to the similarity simulation results, the instability motion modes of the key bearing layer with different horizons under periodic fracture and movement patterns of overburden rock are compared, as shown in Fig. 6. The results of the study (see Fig. 6) show that the fractured rock may turn into two structures, i.e., a "step rock beam" (Fig. 6a) and "masonry-like beam" (Fig. 6b), that is based on the geometrical characteristics and hinged structure of the rock. However, if the mined-out sections are not backfilled, the rock strata below the key bearing layer may collapse.

When the key bearing layer is located within the overburden caving zone. The vertical height range of the relaxation zone is small, and there are few collapsed rocks in the relaxation zone. The fallen rock cannot fill the destruction zone, there is a large free space in the destruction zone. In the broken key bearing layer, rock M and rock N form a "step rock beam" structure, rock N falls completely on the collapsed rocks, while rock M is unstable and moves with the advance of the working face.

When the key bearing layer is located within the overburden fissure zone. The vertical height range of the relaxation zone is big, and there are a large of collapsed rocks in the relaxation zone. Although the fallen rock cannot fill the destruction zone, there is less free space in the destruction zone. In the broken key bearing layer, rock M and rock N form a "step rock beam" structure. Rock N falls completely on the caved coal gangue, rock M rotates with the advance of the mining coal face, the movement form is manifested in downward sliding movement.

## Effect of the instability motion of the key bearing layer on distribution of fissures in the overburden rock

**Figure 7:**
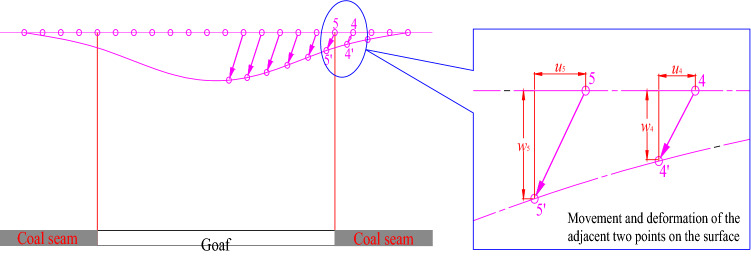
Movement of each point in the overburden rock after shallow coal seam mining.

The fissures in the overburden rock are caused by overburden movement exceeding the limit deformation during coal seam mining^24^. During shallow coal seam mining, the rock strata movement causes vertical and horizontal displacements in the overburden rock, as shown in Fig. 7.

Points 4 and 5 in Fig. 7 are used as an example. The horizontal displacement difference $\Delta u = u_{5} - u_{4}$ between two adjacent points in the overburden rock denotes the horizontal opening of a fissure between the two points. The vertical displacement difference $\Delta w = w_{5} - w_{4}$ between the two points represents the vertical relative displacement of the fissure between the two points.

The coefficient of the displacement difference between two adjacent points is defined as *γ*, then *γ* can be expressed as follows:

2 $$\gamma { = }\frac{{\sqrt {\left( {\Delta u} \right)^{2} { + }\left( {\Delta w} \right)^{2} } }}{\Delta L},$$

where ∆*L* stands for the distance between two adjacent points (m).

The value of *γ* indicates the possibility of fracture between two points. The greater the *γ* value is, the larger the displacement difference between the two points, and the greater the occurrence possibility of fracture.

**Figure 8:**
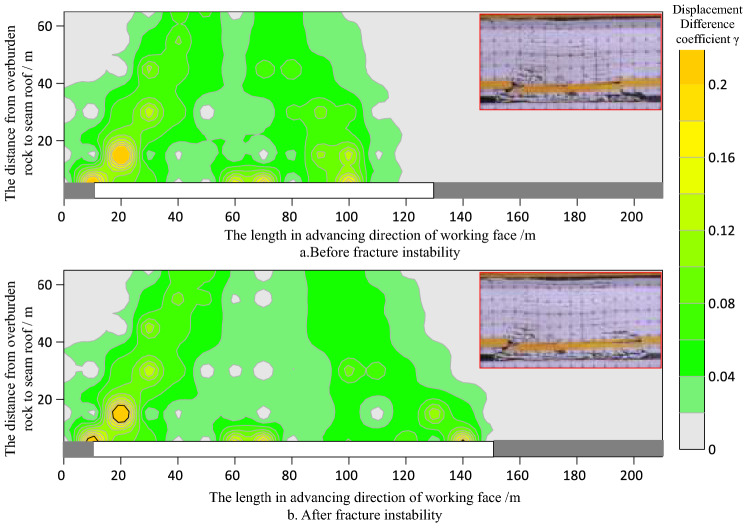
Contours of displacement difference coefficient γ before and after key bearing layer movement in shallow thick seam mining (key bearing layer in caving zone).

**Figure 9:**
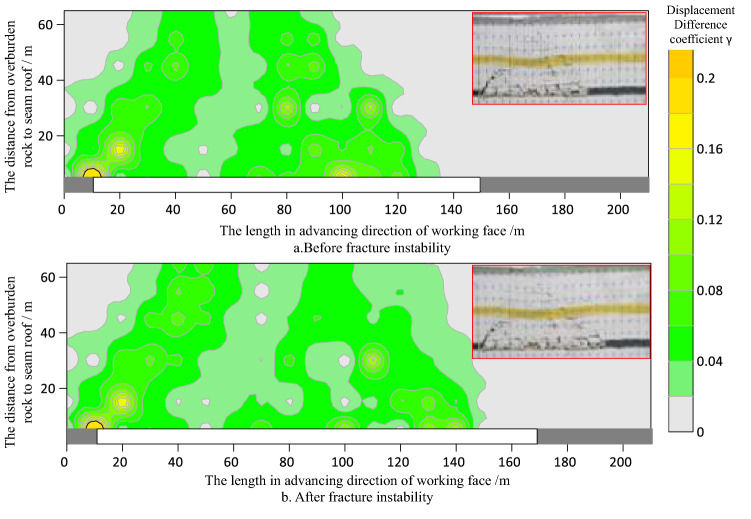
Contours of displacement difference coefficient γ before and after key bearing layer movement in shallow thick seam mining (key bearing layer in fractured zone).

According to the displacement change data of survey points, using “Surfer” drawing software, we plot the contour change cloud map of overburden rock displacement after the failure of the key bearing layer. As shown in Figs. 8 and 9.

Figures 8 and 9 show that as the coal seam is mined, the whole favorable zone for fissures development in the overburden rock extends along the advancing direction of working face in the form of "diagonal stripes".

The position difference coefficient of overburden rock under the key bearing layer is large, which indicates that the fractured rocks within this range are often in an irregular or regular caving state, while the fissures in such rocks are disorderly and unsystematic.

The maximum value of the position difference coefficient of the overlying rock in the key bearing layer moves forward with the working face. The favorable zone for fissure development in the overburden rock above the key bearing layer extends with the instability motion of the key bearing layer.

Influenced by the caving angle of the rock strata, the horizon of the key bearing layer can significantly affect the extension range of the favorable zone for fracture fissure development in overburden rock; that is, when the key bearing layer is within the caving zone, the lag between the working face and favorable zone for fracture fissure development in the overburden rock is small, and when the key bearing layer is within the fissure zone, the lag is large.

## Discussion and limitations of results

Through the experimental study of mining shallow buried coal seam, we have concluded some conclusions. These conclusions may provide some ideas for the study of the development law of overburden fissures caused by coal seam mining, and can also provide some help or suggestions for ground surface damage and ground surface repair caused by coal seam mining.

**Figure 10:**
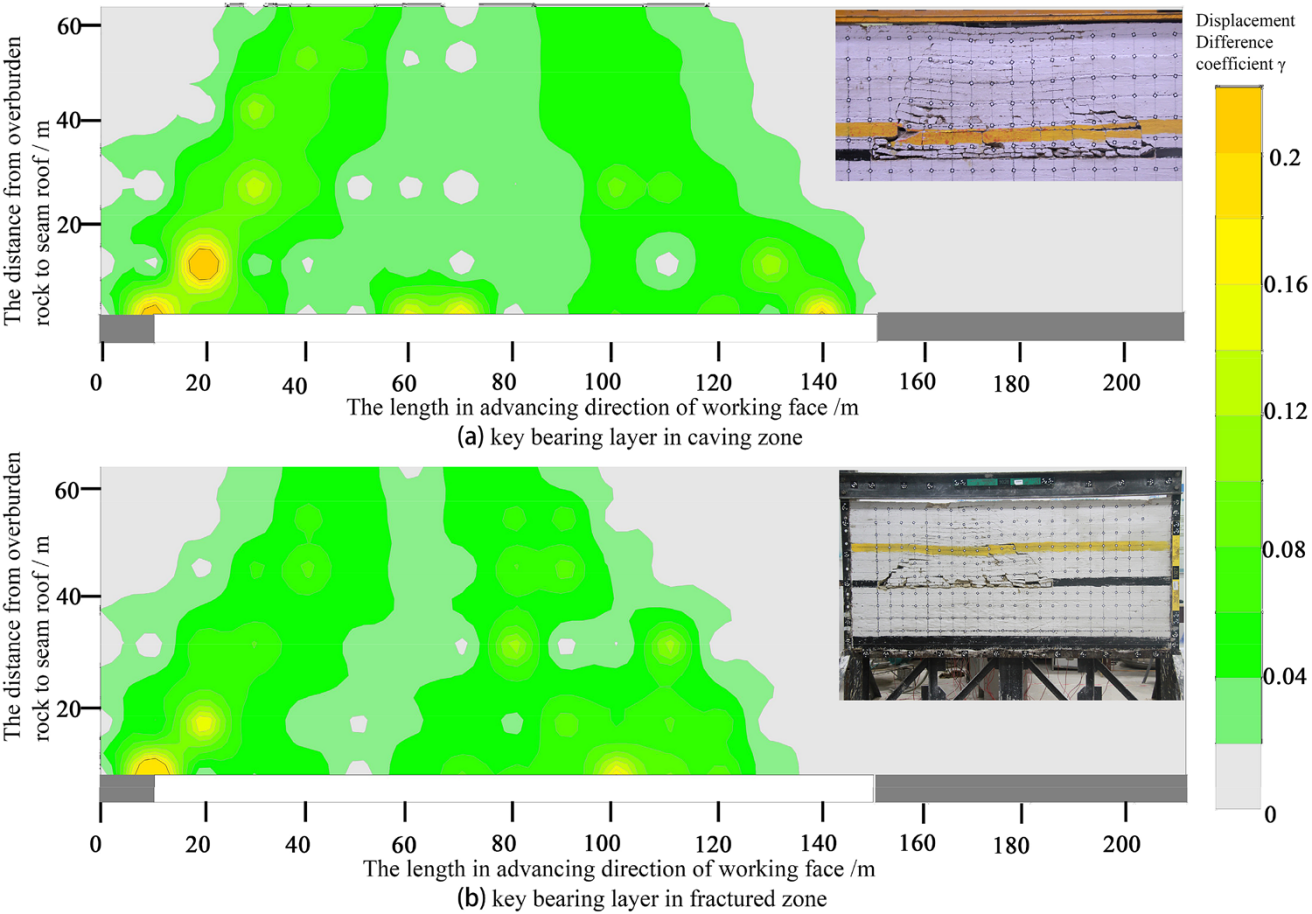
Contours of displacement difference coefficient γ after movement in shallow thick seam mining.

When mining shallowly buried coal seams, the ground surface will be damaged regardless of whether the key bearing layer is in the caving zone. When the key bearing layer is in the caving zone, the damage to the ground surface is larger, and when the key bearing layer is in the fissure zone, the damage to the ground surface is smaller. This conclusion can be drawn from Fig. 10. This shows that the zone of the key bearing layer has an important influence on the land ground surface damage.

In the case of exploitation of deposits at shallow depths of about 100 m, it is very important to protect the ground surface. Compared with studying the breaking laws of rock formations during the mining of shallow coal seams, we should study more relevant methods for protecting the ground surface and repairing the ground surface environment. For example, the method of filling goafs is used to protect the ground surface, and the method of filling ground surface cracks is used to repair the ground surface^27^.

## Conclusion

This paper analyzes the instability movement characteristics of overburden in shallow thick coal seam mining and its influence on the development and distribution of fault fractures. The physical similarity simulation experiment is used to carry out the coal seam mining experiment on the key bearing layer model in different zones. According to the experimental results and the data analysis, the following conclusions can be drawn.

1. By the horizon of key bearing layer, the occurrence conditions of the key bearing layer in the overburden rock of shallow thick seam mining an be classified into two categories, i.e., the key bearing layer within the caving zone and within the fissure zone. After the fracture of the key bearing layer, the fractured rock may turn into two structures, i.e., a "step rock beam" and "masonry-like beam" according to the geometrical characteristics and hinged structure of the rock.
2. The instability motion mode of the key bearing layer within the caving zone is sliding instability. The instability motion mode of the key bearing layer in the fissure zone is rotary instability.
3. The whole favorable zone for fracture fissure development in the overburden rock extends along the advancing direction of the working face in the form of "diagonal stripes", and the favorable zone for fissure development in the overburden rock of the key bearing layer extends with the instability motion of the key bearing layer, while the horizon of the key bearing layer can significantly affect the extension range of the favorable zone for fracture fissure development in overburden rock.

## Data Availability

All data used to support the findings of this study are available from the corresponding author upon request.
